# Markers and Reporters to Reveal the Hierarchy in Heterogeneous Cancer Stem Cells

**DOI:** 10.3389/fcell.2021.668851

**Published:** 2021-06-03

**Authors:** Amrutha Mohan, Reshma Raj Rajan, Gayathri Mohan, Padmaja Kollenchery Puthenveettil, Tessy Thomas Maliekal

**Affiliations:** ^1^Cancer Research, Rajiv Gandhi Centre for Biotechnology, Thiruvananthapuram, India; ^2^Manipal Academy of Higher Education, Manipal, India

**Keywords:** cancer stem cells, phOCT4-EGFP, SORE6-GFP, NANOG-GFP, ALDH1A1-DsRed2, cancer stem cell heterogeneity

## Abstract

A subpopulation within cancer, known as cancer stem cells (CSCs), regulates tumor initiation, chemoresistance, and metastasis. At a closer look, CSCs show functional heterogeneity and hierarchical organization. The present review is an attempt to assign marker profiles to define the functional heterogeneity and hierarchical organization of CSCs, based on a series of single-cell analyses. The evidences show that analogous to stem cell hierarchy, self-renewing Quiescent CSCs give rise to the Progenitor CSCs with limited proliferative capacity, and later to a Progenitor-like CSCs, which differentiates to Proliferating non-CSCs. Functionally, the CSCs can be tumor-initiating cells (TICs), drug-resistant CSCs, or metastasis initiating cells (MICs). Although there are certain marker profiles used to identify CSCs of different cancers, molecules like CD44, CD133, ALDH1A1, ABCG2, and pluripotency markers [Octamer binding transcriptional factor 4 (OCT4), SOX2, and NANOG] are used to mark CSCs of a wide range of cancers, ranging from hematological malignancies to solid tumors. Our analysis of the recent reports showed that a combination of these markers can demarcate the heterogeneous CSCs in solid tumors. Reporter constructs are widely used for easy identification and quantification of marker molecules. In this review, we discuss the suitability of reporters for the widely used CSC markers that can define the heterogeneous CSCs. Since the CSC-specific functions of CD44 and CD133 are regulated at the post-translational level, we do not recommend the reporters for these molecules for the detection of CSCs. A promoter-based reporter for ABCG2 may also be not relevant in CSCs, as the expression of the molecule in cancer is mainly regulated by promoter demethylation. In this context, a dual reporter consisting of one of the pluripotency markers and ALDH1A1 will be useful in marking the heterogeneous CSCs. This system can be easily adapted to high-throughput platforms to screen drugs for eliminating CSCs.

## Introduction

Tumor heterogeneity had been considered as a hallmark of tumors from the very beginning, since the origin of the clonal evolution of cancer. With the tremendous technological advancement over these years, when cells at a single-cell level can be analyzed, it is evident that malignant cells exhibit heterogeneity at the genetic level as well as phenotypic level. Another important feature is the plasticity of these heterogeneous populations, which can be defined as the ability to dynamically switch between these phenotypes. Among these heterogeneous cancer cells, a highly plastic subpopulation with tumor initiation capacity, drug resistance, and metastatic ability, known as cancer stem cells (CSCs), have gained attention as they are responsible for the bad prognosis of the disease (Visvader and Lindeman, 2012). Recent advancement in the field shows that even the CSCs are heterogeneous in nature (Visvader and Lindeman, 2012; Zeng et al., 2014; Turdo et al., 2019; Vander Linden and Corbet, 2019; Velasco-Velazquez et al., 2019; Yang et al., 2020). There are several markers and their combinations used to identify CSCs in a variety of cancers (Table 1). As shown in Table 1, many of the molecules are specific to cancer types, though a few other molecules like CD133, CD44, ABCG2, Aldehyde dehydrogenase (ALDH), and pluripotency markers like octamer binding transcriptional factor 4 (OCT4), SOX2, and NANOG are expressed by a wide variety of cancers, including hematological malignancies and solid tumors. Hence the present review focuses on these molecules and discusses how these molecules and their combinations can be used to demarcate the functionally heterogeneous CSCs in light of the recent advancement in the field. Based on the existing literature, we have gathered a great deal of information for the heterogeneous CSCs in solid tumors. So in the present review, we will focus on solid tumors, with more emphasis on breast cancer.

**Table 1:** CSC markers identified in different cancers.

Markers	Tumor initiating capacity	Drug resistance	Metastasis initiating capacity
CD133	Pancreatic cancer (Banerjee et al., 2014; Nomura et al., 2015)	Colorectal cancer (Yuan et al., 2020) Breast cancer (Nadal et al., 2013) Ovarian carcinoma (Liu et al., 2020) T-cell acute lymphoblastic leukemia (Anbarlou et al., 2015)	Pancreatic cancer (Nomura et al., 2015) Colorectal cancers (Huang et al., 2012a; Fang et al., 2016; Kishikawa et al., 2016) Ovarian cancer (Long et al., 2015)
CD44	Cervical cancer (Feng et al., 2009) Prostate cancer (Ni et al., 2014)	Prostate cancer (Ni et al., 2014) Ovarian cancer (Zhang et al., 2019) Breast cancer (Liu et al., 2017) T-cell acute lymphoblastic leukemia (Hoofd et al., 2016)	Prostate cancer (Ni et al., 2014) Ovarian cancer (Zhang et al., 2019) Oral squamous cell carcinoma (Ortiz et al., 2018) Colorectal cancers (Huang et al., 2012b)
CD123	Acute myeloid leukemia (Abdollahpour-Alitappeh et al., 2018)	Acute myeloid leukemia (Yabushita et al., 2018; Yan et al., 2019)	–
CD26	–	–	Chronic myeloid leukemia (Herrmann et al., 2014)
CD117/c-KIT	Hepatocellular carcinoma (Xu et al., 2018)	Ovarian cancer (Fang et al., 2020)	Hepatocellular carcinoma (Xu et al., 2018)
CD93	–	Chronic myeloid leukemia (Kinstrie et al., 2020)	–
CD9	Acute myeloid leukemia (Liu et al., 2021)	Acute myeloid leukemia (Liu et al., 2021)	–
CD25	–	Acute myeloid leukemia (Allan et al., 2018; Yabushita et al., 2018)	–
ABCG2	Breast cancer (Sicchieri et al., 2015) Colon cancer (Xie et al., 2014)	Colon cancer (Xie et al., 2014) Bladder cancer cells (Roh et al., 2018) Esophageal squamous cancer cells (Huang et al., 2012a) Chronic myeloid leukemia (Jing et al., 2021)	Esophageal squamous cancer cells (Huang et al., 2012a) Hepatocellular carcinoma (Hu et al., 2020) Breast invasive ductal carcinoma (Xiang et al., 2011)
CD49f (ITGA6)	Osteosarcoma (Penfornis et al., 2014) Triple negative breast cancer (Gomez-Miragaya and Gonzalez-Suarez, 2017) Colon cancer (Haraguchi et al., 2013)	Triple negative breast cancer (Gomez-Miragaya and Gonzalez-Suarez, 2017) Ovarian carcinoma (Sedlak et al., 1996)	Breast cancer (Vassilopoulos et al., 2014) Human cervical cancer (Ammothumkandy et al., 2016)
CD66	Human cervical Cancer (Bajaj et al., 2011; Ammothumkandy et al., 2016)	–	Human cervical cancer (Bajaj et al., 2011; Ammothumkandy et al., 2016)
EpCAM/ESA	Hepatocellular carcinoma (Chen et al., 2012) Nasopharyngeal carcinoma (Hoe et al., 2017)	Hepatocellular carcinoma (Chen et al., 2012)	Human colorectal cancer (Chen et al., 2020)
CD90	Lung cancer (Yan et al., 2013) Esophageal squamous cell carcinoma (Tang et al., 2013)	Esophageal squamous cell carcinoma (Tang et al., 2013)	Esophageal squamous cell carcinoma (Tang et al., 2013)
CD166	–	–	Human nasopharyngeal carcinoma (Sun et al., 2019)
LGR5	Breast cancer (Yang et al., 2015)	Colorectal cancer (Hsu et al., 2013)	Colorectal cancer (Valladares-Ayerbes et al., 2012)
OCT4	Hepatocellular carcinoma (Machida, 2018) Gastric cancer (Chen et al., 2019) Breast cancer (Huang et al., 2015)	Gastric cancer (Chen et al., 2019) Breast cancer (Huang et al., 2015) Cholangiocarcinoma (Choodetwattana et al., 2019) Chronic myeloid leukemia (Lettnin et al., 2019) Chronic myeloid leukemia (Xin et al., 2013)	Gastric cancer (Chen et al., 2019) Breast cancer (Litviakov et al., 2020) Colorectal cancer (Roudi et al., 2020)
SOX2	Hepatocellular carcinoma (Machida, 2018) Gastric cancer (Chen et al., 2019)	Gastric cancer (Chen et al., 2019) Breast cancer (Guan and Guan, 2020) Melanoma (Si et al., 2020) Chronic myeloid leukemia (Xin et al., 2013)	Gastric cancer (Chen et al., 2019) Breast cancer (Guan and Guan, 2020; Xiao et al., 2020)
NANOG	Hepatocellular carcinoma (Machida, 2018) Breast cancer (Huang et al., 2015)	Breast cancer (Huang et al., 2015) Esophageal squamous cancer (Deng et al., 2017) Chronic myeloid leukemia (Xin et al., 2013)	Urinary bladder cancer (Gawlik-Rzemieniewska et al., 2016)
ALDH1A1	Human pancreatic adenocarcinoma (Kim et al., 2011) Prostate cancer (Nishida et al., 2012)	Ovarian cancer (Januchowski et al., 2016) Multiple myeloma (Yang et al., 2014) Acute lymphoblastic leukemia (Ahlers et al., 2014)	Breast cancer (Wang et al., 2018) Papillary thyroid carcinoma (Yue et al., 2015)

The tumor microenvironment surrounding CSCs or a “CSC niche” plays a critical role in regulating the high plasticity exhibited by CSCs subpopulations (Thankamony et al., 2020). Several factors existing in the CSC niche, including hypoxia, acidic pH, cancer-associated fibroblasts (CAFs), and altered cytokine levels, contribute to the characteristics acquired by CSCs (Saygin et al., 2019). Thus even in a single tumor, there could be heterogeneity in the CSCs, depending on the niche they reside (Visvader and Lindeman, 2012). The existence of a dormant CSC population as well as proliferative CSCs is known in many cancers, and they show different levels of differentiation (Bliss et al., 2016; Shanmugam et al., 2019). Although CSCs might have properties like tumor initiation capacity, drug resistance, and/or metastatic ability, a single CSC at a given time point may not show all the three properties. But all the widely-used markers identify CSCs enriched for tumor initiation potential, drug resistance, and metastasis initiating efficiency (Table 1). In other words, these markers identify a group of CSCs exhibiting different characteristics and levels of differentiation. Though the functional characterization assign CSCs to different hierarchical groups akin to stem cell hierarchy, it was difficult to physically separate them because of the inadequacy of markers (Boesch et al., 2016) until recent developments in the CSC field using single-cell analyses. A detailed analysis revealing the phenotype of each CSC-subpopulation can reveal a marker profile that can demarcate the subpopulations of CSCs.

## Heterogeneity and Hierarchy in CSCs

A hallmark of CSCs is their potential to generate phenotypically and functionally heterogeneous populations, as a result of metabolic reprogramming and a series of symmetric and asymmetric cell divisions. These subpopulations show the ability to interconvert, or they exhibit plasticity, which is the outcome of a reprograming initiated by stemness signals present in the “CSC niche” (Thankamony et al., 2020). The CSCs which are dormant can acquire characteristics including drug resistance and metastatic initiation potential. Additionally, this dormant population can acquire proliferative capacity to facilitate differentiation. Recent advances in the single-cell-based technologies, such as single-cell DNA/RNA-Sequencing, mass cytometry (CyTOF), next-generation fluorescence flow cytometry, and “imaging mass cytometry” platform, help us to understand how functional heterogeneity is reflected by phenotypic heterogeneity (Akrap et al., 2016; Puram et al., 2017; Colacino et al., 2018; Sharma et al., 2018; Prieto-Vila et al., 2019; Taverna et al., 2020; Frank et al., 2021; Gonzalez Castro et al., 2021).

**Figure 1:**
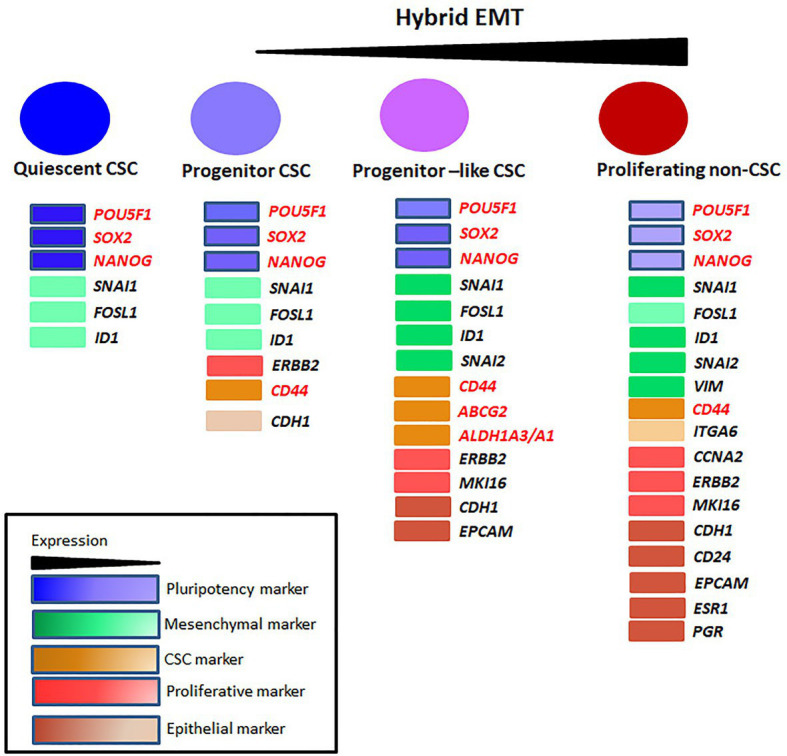
The heterogeneity in cancer stem cells (CSCs). The different populations identified in breast CSCs and their markers are represented. The non-dividing Quiescent population, Progenitor population, Progenitor-like population, and the differentiated non-CSC population express a gradient of pluripotent, mesenchymal/epithelial and proliferative markers. The Quiescent population expresses a high level of pluripotent transcriptional factors and a moderate level of mesenchymal markers. With differentiation, the level of pluripotent markers comes down. The Progenitor population starts to express proliferative markers, CD44 and epithelial markers in very low levels. The Progenitor-like population co-expresses mesenchymal and epithelial markers manifesting a gradual increase in hybrid-epithelial-mesenchymal transition (EMT) phenotype. This population also expresses CSC markers ALDH1A1/A3 and ABCG2 contributing to drug-resistance. Non-CSCs lose the expression of pluripotent and other CSC markers except for CD44. The proliferative markers, epithelial markers, and mesenchymal markers are at maximum expression level in this population. This population can metastasize, but cannot initiate tumor until they dedifferentiate into CSCs.

Hypothetically, distinct states of long-term CSCs and committed Progenitor cells exist in CSC pool. According to this, a Quiescent CSC is equivalent to an adult stem cell, which gets converted to a Progenitor CSC, probably by an asymmetrical cell division. This cell further undergoes proliferation to generate a subpopulation of Progenitor-like population, still possessing CSC characteristics. Further divisions of these cells generate proliferating cells, devoid of CSC characteristics (Proliferating non-CSCs; Figure 1). Later, these non-CSCs might lose the proliferative capacity, and can attain dormancy. The single-cell analyses have provided some evidences to show the hierarchy in CSCs (Akrap et al., 2016; Colacino et al., 2018; Sharma et al., 2018). Yet, a direct proof for this differentiation in a biological assay is still lacking. We need a clear understanding of the marker profiles for each of the different stages of differentiation to trace the hierarchy in the stem-like CSCs. If we can generate reporter constructs to mark each of the stages, we can trace the fate of CSCs. In the following sections, we describe the hierarchical organization of the heterogeneous CSCs and their marker profiles.

### Functional Heterogeneity in CSCs

**Figure 2:**
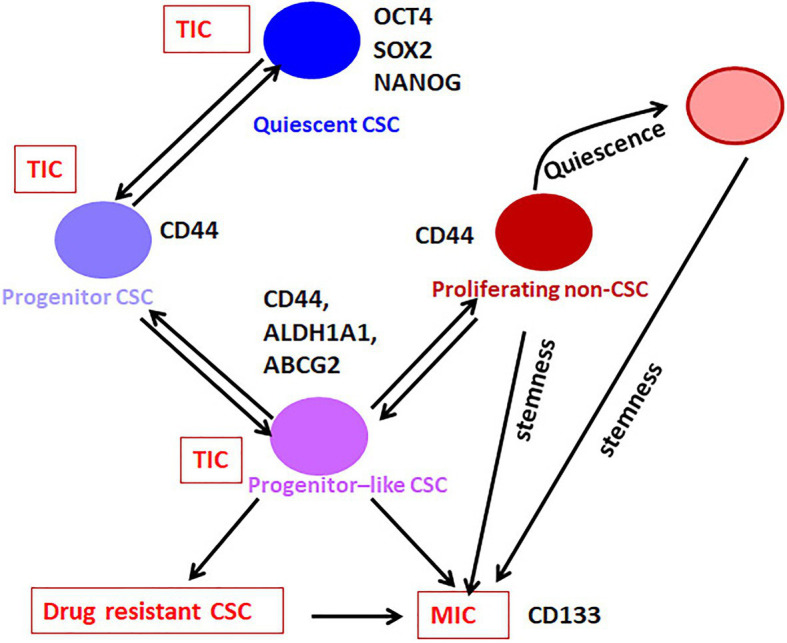
The hierarchy in CSCs. Our analysis on the available literature on single-cell analyses conducted in breast cancer has revealed that the expression of pluripotency markers without CD44 marks the Quiescent CSC, while CD44 along with moderate expression of pluripotency markers define the Progenitor CSC population. These cells gradually lose the expression of pluripotency markers and gain the expression of ABCG2 and ALDH1A1 to get converted to Progenitor-like CSC population. According to the niche factors, these cells can be converted to the drug-resistant-CSCs or metastasis initiating cells (MICs). When this Progenitor-like CSC population loses all the CSC markers except CD44, they are converted to the Proliferating non-CSCs. The Quiescent cells, Progenitor cells, and Progenitor-like cells are considered as CSCs or tumor-initiating cells (TICs), which exhibit tumor initiation potential. The Progenitor like population having hybrid EMT phenotype might be the drug-resistant CSCs and MICs. The proliferating population with hybrid EMT might be highly metastatic, and they can remain dormant at distant sites. These cells are the seeds of recurrence at a different site. They might initiate tumor if they acquire stemness depending on the metastatic niche.

Hypoxia is one of the critical cues that initiate reprogramming in cancer cells to escape unfavorable conditions like nutrient deprivation or therapy by inducing dormancy/quiescence or epithelial-mesenchymal transition (EMT), which in turn help them to acquire invasive capacity, metastatic ability, and chemoresistance (Xiong et al., 2020). Thus a hypoxia-mediated reprogramming can generate heterogeneous CSCs. The functional heterogeneity of CSCs is exhibited as Quiescent CSCs, tumor-initiating cells (TICs), metastasis-initiating cells (MICs), and drug-resistant CSCs (Figure 2). The Quiescent CSCs are marked with low metabolic rate, slow cell division, and a shift of oxidative respiration to glycolysis, manifesting the Warburg effect (Yuen et al., 2016). These slow cycling cells can survive in extreme conditions and can overcome chemotherapy because a majority of the chemotherapeutic drugs are targeting dividing cells. Once they survive the adverse condition, they try to escape from that site by inducing EMT. During EMT, cells lose their polarity, cell-cell adhesion and undergo cell cycle arrest to gain mesenchymal properties like increased motility. EMT programs bring about changes in the cell shape, cytoskeleton, and secretome profiles, which is brought about by the tight regulation of a set of EMT genes and transcription factors (Celia-Terrassa and Jolly, 2020). This process might have three progressive stages, where cells can have EMT with more epithelial nature or a hybrid EMT with equal epithelial and mesenchymal nature or EMT with more mesenchymal characteristics (Celia-Terrassa and Jolly, 2020). Though EMT was shown to induce stemness in cancer cells (Mani et al., 2008), the stemness property is limited to the population showing hybrid EMT (Celia-Terrassa and Jolly, 2020). Hence in the CSC context, the cells with the hybrid EMT will have increased invasiveness, motility, and CSC characteristics like tumor-initiating property. These cells are the MICs. The other two populations are not able to establish metastasis as the more epithelial EMT cells lack motility and the extreme mesenchymal cells are devoid of tumor-initiating properties (Celia-Terrassa and Jolly, 2020). The metastatic cells with extreme mesenchymal characteristics can remain dormant at distant sites and can undergo mesenchymal-epithelial transition to acquire hybrid phenotype and tumor initiation potential (Weidenfeld and Barkan, 2018). Apart from the metastatic ability, EMT can regulate chemoresistance also to generate drug-resistant CSCs. The EMT transcription factors like TWIST and SNAI1 are reported to regulate chemoresistance through the upregulation of drug metabolizing enzymes and drug efflux molecules (Rodriguez-Aznar et al., 2019). Additionally, the EMT factors like ZEB1, ZNF281, and SNAI2 modulate DNA damage and DNA repair to impart chemoresistance (Rodriguez-Aznar et al., 2019).

When the heterogeneity of populations in the context of drug resistance was analyzed at a single-cell resolution in breast cancer cells, expansion of a pre-existing subpopulation was identified (Prieto-Vila et al., 2019). The parental population had a high expression of epithelial markers like *CDH1* and *PGR* as well as a moderate expression of mesenchymal markers *VIM* and *SNAI1* denoting the hybrid EMT phenotype (Prieto-Vila et al., 2019). The stemness markers like *ABCG2*, *SOX2*, *ITGA6*, and *CAV1/2* were also high in this subpopulation. Notably, there was some amount of proliferative markers also present in this population, so that they could proliferate. When these cells were exposed to docetaxel, the resistant population emerged retained a majority of the markers except *ITGA6*, *SNAI1*, and the proliferative markers that showed a diminishing pattern (Prieto-Vila et al., 2019). So, probably these cells represent the slow cycling CSCs that acquired *ABCG2* and hybrid EMT. When the mechanism of drug resistance induced by cisplatin was analyzed in oral cancer cell lines, it was noted that drug resistance could be achieved by pre-existing pools of resistant cells or by reprogramming as an adaptive mechanism. They showed that cisplatin resistance could be achieved by either the selection of SOX2^+^/CDH1^+^ population or by reprogramming to mesenchymal SOX9^+^/VIM^+^ population (Sharma et al., 2018).

Epithelial-mesenchymal transition, more specifically the hybrid EMT, is the prerequisite for metastasis. In this state, the cells express both epithelial and mesenchymal markers. Induction of hybrid EMT in CSCs might convert them to MICs. A single-cell RNA-Seq analysis conducted in head and neck squamous cell carcinoma cells showed that the classical signature genes for hybrid EMT are *EPCAM*, *VIM*, *TGFBI*, and *SNAI2* (Puram et al., 2017). Single-cell RNA-Seq analysis conducted with breast cancer CSCs showed a population with hybrid EMT and CSC markers *CD44*, *ABCG2*, and *ALDH1A1/3*, which might mark the MICs (Akrap et al., 2016). At the same time, there was another hybrid EMT population lacking *ABCG2* and *ALDH1A1/3*, but with *ITGA6*, probably representing metastatic cells without tumor-initiating efficiency (Figure 1; Akrap et al., 2016). In majority of the studies, they are treated as proliferating non-CSC cells, but they need to be considered separately, because they exhibit high hybrid EMT. Here, we have to recall that majority of the non-CSC population do not exhibit high hybrid EMT. We have reported a fraction of ITGA6^+^ cells exhibiting epithelial characteristics involved in metastasis (Ammothumkandy et al., 2016). The recent evidences suggest that the proliferative non-CSCs can acquire resistance and aid in metastasis after stemness induction depending on the CSC niche, probably explaining why the ITGA6^+^ cells showed metastatic ability. In a proteomic analysis, CD133 was observed in hybrid EMT phenotype, which is shown to be important for metastatic cells (Taverna et al., 2020). Whether the CD133 expression is exclusive to the Progenitor-like population is not yet evident. At least in colon cancer, ITGA6 expressing cells fall into CD44^+^/CD133^+^ cell fraction, probably representing the metastatic non-CSCs (Haraguchi et al., 2013).

When we analyze the level of differentiation, these functionally different CSCs fall into distinct stages. The Quiescent population resembles the G_0_-arrested stem cells, while the drug-resistant CSCs and MICs have limited proliferative capacity and self-renewal ability, similar to the Progenitor-like population. At the same time, the majority of the cancer cells, which do not have self-renewal ability but possess proliferative capacity, mimics the differentiated population. Recent evidences show that these subsets can be differentially identified using the marker profiles, and they show a hierarchical organization.

### Hierarchical Organization in CSCs

Cancer stem cells are enriched by different methods, like spheroid culture (showing anoikis resistance), sorting using markers of CSCs, sorting based on label retention (indicating slow cycling population) or by inducing hypoxia. An elegant study in which breast cancer CSCs were enriched by these three methods and subjected to single-cell RNA-Seq has shed light on the phenotypic heterogeneity exhibited by CSCs (Akrap et al., 2016). They identified a Quiescent stem-like population, a Progenitor population, a more proliferative Progenitor-like population and a proliferating population (Figure 1). Among these, the subpopulations except the proliferating cells are generally considered as CSCs (Akrap et al., 2016).

When cells are exposed to hypoxia, very few cells are Quiescent, and Progenitor-like population predominates. But anoikis resistance enriches the Quiescent cells, Progenitor cells, and Progenitor-like cells in equal proportions. When slow cycling cells were picked up by label-retention, the majority of the population was the Progenitor population, followed by the Progenitor-like population and Quiescent population (Akrap et al., 2016). In this case, when cells were analyzed, the Quiescent population had low expression of all the genes including pluripotency genes compared to the Progenitor population (Akrap et al., 2016). So it suggests that the Quiescent population itself might have two populations, one with pluripotent markers denoting the stem-like CSCs and the other without pluripotent markers, which might be the non-CSCs.

Except for the pluripotency markers, all the other markers were low in the Quiescent stem-like population compared to other populations, although mesenchymal markers *SNAI1* and *FOSL1* were expressed in these cells (Akrap et al., 2016; Figure 1). These cells started expressing more epithelial markers like *CDH1* along with proliferative marker *ERBB2* to get converted to the Progenitor population expressing *CD44*. At this stage, the expressions of pluripotency markers were progressively lost (Akrap et al., 2016). Since the later differentiation process requires a series of cell divisions, they started expressing more proliferative markers like *MKI67* and acquire a hybrid EMT phenotype with the expression of *EPCAM*, *SNAI2*, and *ID1*. At this stage, they expressed stemness markers like *ALDH1A3* and *ABCG2*. This population is the proliferative Progenitor-like population. These cells proliferated further and expressed more proliferative markers and increased the expression of epithelial markers along with mesenchymal marker *VIM*. At this stage, these proliferating cells expressed a modest amount of *POU5F1*, *NANOG*, and *ITGA6* but started losing *CD44*, *ABCG2*, and *ALDH1A3* (Akrap et al., 2016).

At present, CSCs can be considered as a heterogeneous population that has tumor initiation potential comprising of stem-like Quiescent cells, Progenitor cells, and Progenitor-like cells, which progressively lose self-renewal capacity and acquire proliferative capacity (Figure 2). From the Progenitor cell stage, cells start acquiring hybrid EMT and there is a population with the highest hybrid EMT, but lacks tumor initiation potential. All these populations are important with respect to cancer progression, metastasis, and chemoresistance. At the marker profile, the Quiescent stem-like population expresses the pluripotency markers, while the Progenitor population in breast cancer cells can be marked by CD44 with diminished pluripotency markers and without other CSC markers like ALDH1A1/3 or ABCG2 (Figure 1). When the cells attain the Progenitor-like phenotype, they express all these markers. The proliferating cells do not express CSC markers except CD44 and ITGA6. Thus, the stem-like Quiescent cells express only pluripotency markers at high levels. A moderate amount of pluripotency markers and CD44 marks the Progenitor population, while high expression of ALDH1A1/3, ABCG2, CD44, and low amount of pluripotency marker denote Progenitor-like population. At the same time, CD44 and ITGA6 mark the proliferative non-CSC population.

Taken together, there are certain marker profiles that can demarcate the heterogeneous CSCs. It is evident that many of these molecules were used as CSC markers for different cancers, as shown in Table 1. The currently used markers for CSCs are the molecules regulating CSC characteristics, which are expressed specifically in this population. They include several cell surface molecules, pluripotency markers, and other molecules like ALDH (Table 1). In the following sections, we give a short account of some of the consistently used markers for CSCs, and discuss how they regulate different properties of CSCs. Special emphasis is given to the molecules used for making reporters of CSCs.

## Markers of CSCs

### Cell Surface Molecules as CSC Markers

Cell surface markers are an important class comprising receptors or ligands, which mediate signaling cascades regulating tumorigenic properties, cell adhesion molecules, and transporter molecules like ATP binding cassette (ABC) transporters. Some of the most important receptors include CD133 and CD44, while ABCG2 is a vital drug transporter that marks CSCs.

### CD133

CD133 (Prominin 1) is a penta-transmembrane surface glycoprotein coded by the gene *PROM1*. Following two papers reporting CD133 as a marker for CSCs in colorectal cancer (O’Brien et al., 2007; Ricci-Vitiani et al., 2007), there was a surge of reports showing CD133 as a marker for CSCs of different origin. Consistent with that, there are reports showing CD133^+^ subset’s association with chemo-resistance, metastasis, and poor prognosis (Irollo and Pirozzi, 2013). Hence, initially it appeared that this molecule will be a universal marker for CSCs. But as the field evolved it was evident that discrepancies exist even in the same cancer. There are accumulating evidences that question the CSC nature of CD133^+^ population in several cancers, including colon cancer, glioma, and lung cancer, as CD133^−^ population initiated long term tumors *in vivo* in these studies (Shmelkov et al., 2008; Irollo and Pirozzi, 2013).

There are several factors that determine the suitability of a molecule as a marker. The method of detection is critical in the case of CD133. It is generally detected using an antibody raised against AC133 epitope, which is exposed only after glycosylation of the region. So the antibody recognizes only the glycosylated form of the molecule. A comparative study of this antibody and another antibody that recognizes the non-glycosylated form showed that only the glycosylation is negatively regulated with differentiation (Florek et al., 2005). In colon cancer, the reduction in population expressing the AC133 epitope and loss of clonogenicity upon differentiation of CSCs did not correspond to a reduction of CD133 promoter activity or its expression at mRNA or protein level (Kemper et al., 2010). Thus it is evident that only the glycosylated CD133 is the actual marker for stemness. Since reporter constructs cannot reflect the post-transcriptional and post-translational modifications, a reporter construct for this molecule may not be useful in identifying CSCs, which was actually observed. A knock-in lacZ reporter mouse (CD133lacZ/+) was generated in which the expression of lacZ is driven by the endogenous CD133 promoter. Using this reporter, they showed that CD133 is ubiquitously expressed in colonic epithelium. A modified form of this murine model showed that in murine colon adenocarcinoma, all the cells except stromal cells and infiltrating cells are CD133^+^(Shmelkov et al., 2008).

**Figure 3:**
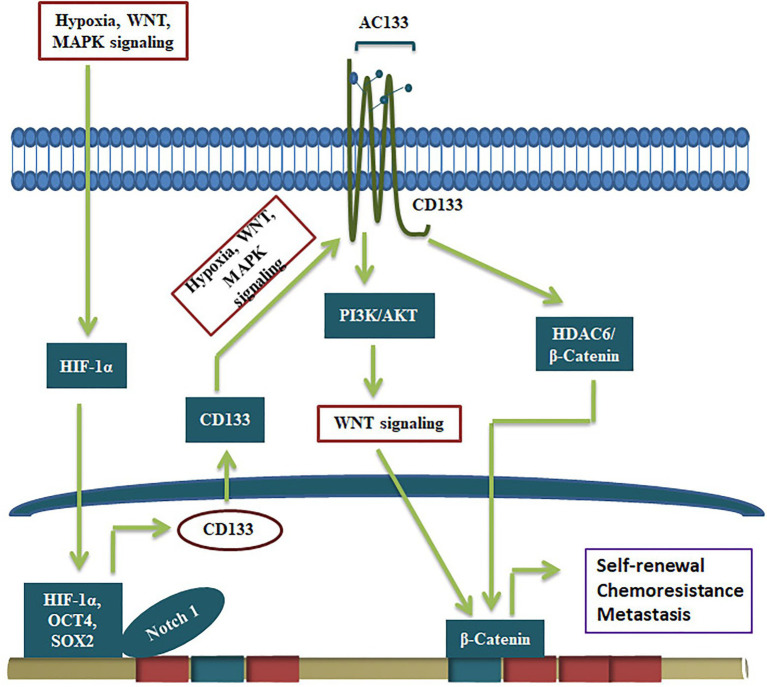
CD133 in the regulation of CSCs. The expression of CD133 and its conversion to active form containing AC133 epitope by glycosylation is regulated by upstream signals like hypoxia and pathways like MAPK and WNT. The active CD133 primarily regulate WNT/β-catenin pathway to drive self-renewal.

Even though CD133 is not a suitable marker to make reporters for CSCs, the glycosylated functional CD133 imparts several CSC characteristics to CSCs (Figure 3). The significance of CD133 signaling was not unraveled for a long time. A report that showed CD133’s interaction with cholesterol to act as an organizer for membrane topology was the first report that suggested its role in signaling (Florek et al., 2005). However, its relevance in stem cell biology was not unraveled until a physical interaction of CD133 to p85 subunit of PI3K was shown to activate its catalytic subunit p110 (Wei et al., 2013). Further, the CD133/PI3K/AKT pathway is shown to activate WNT signaling to drive glioblastoma tumor-initiating cells (Manoranjan et al., 2020). CD133 can regulate WNT signaling by another mode, where it forms a ternary complex with HDAC6 and β-catenin, stabilizing β-catenin, resulting in the activation of WNT/β-catenin signaling (Figure 3). Further, the CD133 expression, the resultant WNT activation and the increase in the CSC properties are shown to be regulated by several other self-renewal pathways. Hypoxia, a known stemness-inducing factor, is shown to increase CD133 expression at the RNA level and enhances the glycosylation and the AC133 epitope (Wu and Chu, 2019). The expression and surface localization of CD133 is activated by MAPK/ERK, HIF-1α, Wnt/β-catenin, and JAK/STAT3 (Gzil et al., 2019). Consistent with that, the depletion of CD133 leads to loss of CSC properties and conversely, its over-expression leads to a gain of stemness characteristics (Chen et al., 2011; Simbulan-Rosenthal et al., 2019).

Single-cell sequencing of CD133^+^ cells from colorectal cancer cells from a patient has revealed the heterogeneity among the population with respect to copy number and mutational profile (Min et al., 2020). When CD133^+^ cells were compared with CD133^−^ cells, bulk tumor cells, and tumor cells from metastatic sites, the heterogeneity among CD133^+^ cells was revealed. However, mutations of at least three genes among the seven genes (*RNF144A*, *PAK2*, *PARP4*, *ADAM21*, *HYDIN*, *KRT38*, and *CELSR1*) was consistently observed in CD133^+^ cells, while all the seven mutations were observed in metastatic tumor cells, suggesting that a subset of CD133^+^ cells are primarily responsible for establishing metastatic lesions (Min et al., 2020). There was another study conducted in parallel using single-cell proteomic profiling with lung cancer samples, where a CyTOF panel of 21 antibodies was designed to probe lung cancer cells. They observed a higher level of CD133 in comparison to other stemness markers (OCT3/4, NANOG, CD44, and ALDH1A1) in a subpopulation that exhibited a hybrid nature of epithelial/mesenchymal plasticity (Taverna et al., 2020). Recently it is postulated that epithelial-mesenchymal plasticity is the driving force of CSCs during metastasis (Celia-Terrassa and Jolly, 2020). So, the molecular evaluation to the resolution of single cells reveal that co-expression of CD133, OCT3/4, NANOG, CD44, and ALDH1A1 might mark cells with CSC characteristics, while increased expression of CD133 is observed in hybrid EMT phenotype, which is a pre-requisite for metastasis.

### CD44

CD44 is a cell surface adhesion receptor that sense, integrate, and transduce extracellular matrix signals to cells, and regulate several genes resulting in changes in cell behavior. The expression of CD44 is reported in a wide variety of epithelia of both squamous and glandular origin along with their neoplastic counterparts. In this context, we need to examine the relevance of this molecule as a CSC marker. Apart from the standard CD44 isoform (CD44s ∼85 kDa), at least nine variants formed by alternative splicing (CD44v) are identified in humans. Using specific antibodies, it was shown that CD44s and CD44-9v are ubiquitously expressed in all epithelial tissues but their expression in glandular tissue was restricted to the basal layer (Mackay et al., 1994). Their observations suggest that CD44v is expressed by stem/progenitor cells of both squamous and glandular epithelia. When they differentiate, the expression of CD44v is retained in squamous epithelium till terminal differentiation, while its expression is lost during differentiation of glandular epithelium. Later, several other reports reinforced this notion in different epithelia (Ylagan et al., 2000). So when a primitive marker is re-expressed in cancer cells of glandular origin, it marks the CSCs as observed in many adenocarcinoma cells (Table 1). Consistent with that, intestinal stem cells isolated using Lrg5 marker from mouse and familial adenomatous polyposis samples express CD44v, but lack the standard CD44s isoform (Zeilstra et al., 2014). Also, in experiments using knock-in mice expressing either CD44v4-10 or CD44s, it was demonstrated that the CD44v isoform, but not CD44s, promotes adenoma initiation in Apc (Min/+) mice (Zeilstra et al., 2014). At the same time, in cancers of squamous cell origin, CSCs are a subpopulation within CD44s/CD44v expressing cells (Table 1). Further, the expression of CD44, both CD44s and CD44v, is associated with poor prognosis in a majority of cancers (Thapa and Wilson, 2016), since CD44 signaling leads to EMT, metastasis, and resistance to therapy.

CD44 is a non-kinase glycoprotein membrane receptor for hyaluronic acid (HA), osteopontin (OPN), chondroitin, collagen, fibronectin, and sulphated proteoglycans. Even though it is not clearly understood the specificity of variants to different ligands, it is well established that when CD44 binds to HA, it leads to a conformational change in the CD44 molecule allowing it to act as a co-receptor or adaptor for other signaling molecules (Bourguignon, 2019). There are many reports showing that the expressions of stemness markers like SOX2, NANOG, and OCT4 are high in CD44^+^ population (Gzil et al., 2019). Though the signaling cascades downstream of CD44 is not well-characterized, it is known that CD44 activates different signaling pathways like Rho GTPases, Ras-MAPK, and PI3K/AKT pathways to regulate different tumor properties (Al-Othman et al., 2020). In the CSC context, it is reported that the interaction of HA with CD44 leads to NANOG-STAT3 activation in ovarian cancer cells, making it a potential molecule for marking self-renewing population (Fang and Kitamura, 2018).

**Figure 4:**
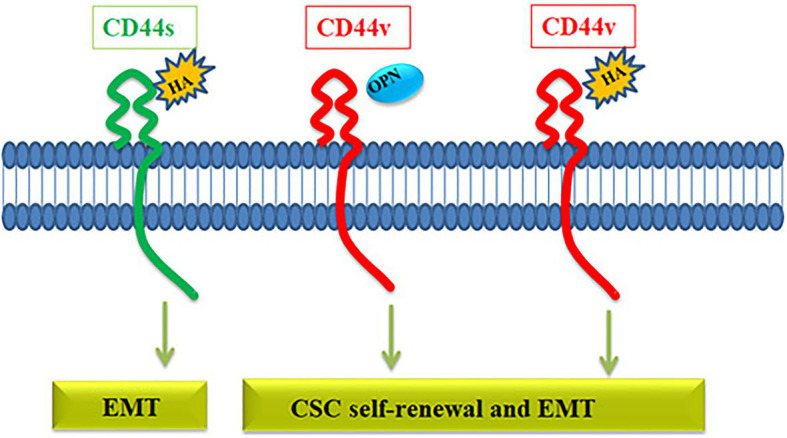
The role of CD44 in the regulation of CSC properties. The CD44 gene gives rise to CD44s variant or CD44v variant. CD44s interacts with hyaluronic acid (HA) activating pathways that induce EMT. HA can also bind to CD44v receptor and activate EMT and self-renewal through various pathways. Another ligand osteopontin (OPN) binds specifically to CD44v, which induces EMT and self-renewal.

The relevance of the variants and the choice of the ligand might be critical in defining the downstream signaling of CD44. Among the variants, it is almost clear that CD44v is more critical for maintaining the CSC population than CD44s (Figure 4). Even though several ligands are reported for CD44, the well-characterized ones in context to cancer are HA and OPN. HA can bind to all forms of CD44, while OPN does not bind to CD44s but binds to the CD44v, specifically CD44v6 (Katagiri et al., 1999). Both HA and OPN are reported to regulate CSCs in different contexts. HA, directly and indirectly, affects CSC self-renewal by influencing the behavior of both cancer and stromal cells by regulating EMT (Chanmee et al., 2015). When HA mediates the signaling through CD44v, a set of miRNAs including, miR-21, miR-302, and miR-10b are upregulated that regulate different CSC properties (Bourguignon, 2019). So, HA can mediate signaling through all CD44 forms and regulate EMT, which is a determinant of stemness, invasion, and metastasis, explaining how overexpression of CD44 correlates with poor prognosis. OPN, a possible ligand for CD44v variants, is reported to be enriched in glial tumors and the OPN-CD44 axis promotes CSCs in glioma (Lamour et al., 2015). Further analysis has shown that OPN-silenced glioma-initiating cells are unable to grow as spheres, and lose the expression of SOX2, OCT3/4, and NANOG. Further, AKT/mTOR/p70S6K pathway was identified as the main signaling pathway triggered by OPN in glioma-initiating cells (Lamour et al., 2015). Collectively, a preference of the ligand present in the microenvironment might be a critical regulator in determining the cell fate of CD44 expressing cells. If HA is expressed, all the CD44 expressing cells respond to it by acquiring EMT, and if the ligand switches to OPN, only the CD44v subsets respond to it by showing CSC properties. Consistently, hypoxia, a known regulator of stemness, regulates the expression of OPN without affecting the expression of CD44 variants in colorectal cancer cells (Wohlleben et al., 2018). All these facts point out that CD44 expression *per se* is not a marker of CSCs, but the expression of specific variants and the choice of ligands might define the CSCs. Thus reporter for this molecule may not be relevant in CSC detection.

Single-cell RNA profiling of CSCs from breast cancer cells, where CD44 is considered as a CSC marker, has revealed some significant information regarding CD44 expression in the context of hypoxia and the hierarchy of CSCs. When CSCs were enriched by either anoikis resistance, label retention or induction of hypoxia, there were subpopulations within CSCs, a Quiescent population, a Progenitor population and a more proliferative Progenitor-like population, which eventually differentiated to bulk tumor cells. This Quiescent population had less *CD44*, which was increased in the Progenitor-like, more proliferative CSCs (Akrap et al., 2016). In the differentiated bulk cells, the expression of *CD44* was less compared to the Progenitor population, but high compared to the Quiescent population. Yet this conclusion is not final because the expression of variants had not been taken into account in this analysis.

### ABCG2

ABCG2 is a member of the ABC transporters, which function as membrane transporters, ion channels, or receptors to pump a wide variety of endogenous and exogenous compounds out of cells (Polgar et al., 2008). It confers side population phenotype and is considered as a universal marker of stem cells. The high expression of ABCG2 is observed in various malignancies, and is usually associated with poor prognosis (Ding et al., 2010; Table 1). One of the possible roles suggested for ABCG2 is pumping the differentiation factors out of the cells, so that the cell retains the self-renewal capacity (Sabnis et al., 2017). The high expression of ABCG2 in CSCs is regulated by promoter demethylation, histone modification, and transcriptional upregulation by different self-renewal pathways (Ding et al., 2010; Stacy et al., 2013). Another factor important in clinical relevance is the single nucleotide polymorphism of ABCG2, which critically regulates the pharmacokinetics of different drugs (Heyes et al., 2018).

Even though ABCG2 is reported to be active in embryonic stem cells and other Quiescent stem cells, it is enriched more in the Progenitor-like population than in the Quiescent population, when analyzed by single-cell RNA-Seq in CSCs of breast cancer (Akrap et al., 2016). Anoikis-resistance and hypoxic conditions, which induce Quiescent cells, increase the expression of ABCG2, as reported before (Ni et al., 2010; Prabavathy et al., 2018), but the expression is restricted to the Progenitor-like subpopulation in these conditions (Akrap et al., 2016). Among the stem cell markers used, *CD44* and *ABCG2* were regulated in a similar pattern, and were enriched in the Progenitor-like population.

### Pluripotency Transcription Factors That Mark CSCs

**Figure 5:**
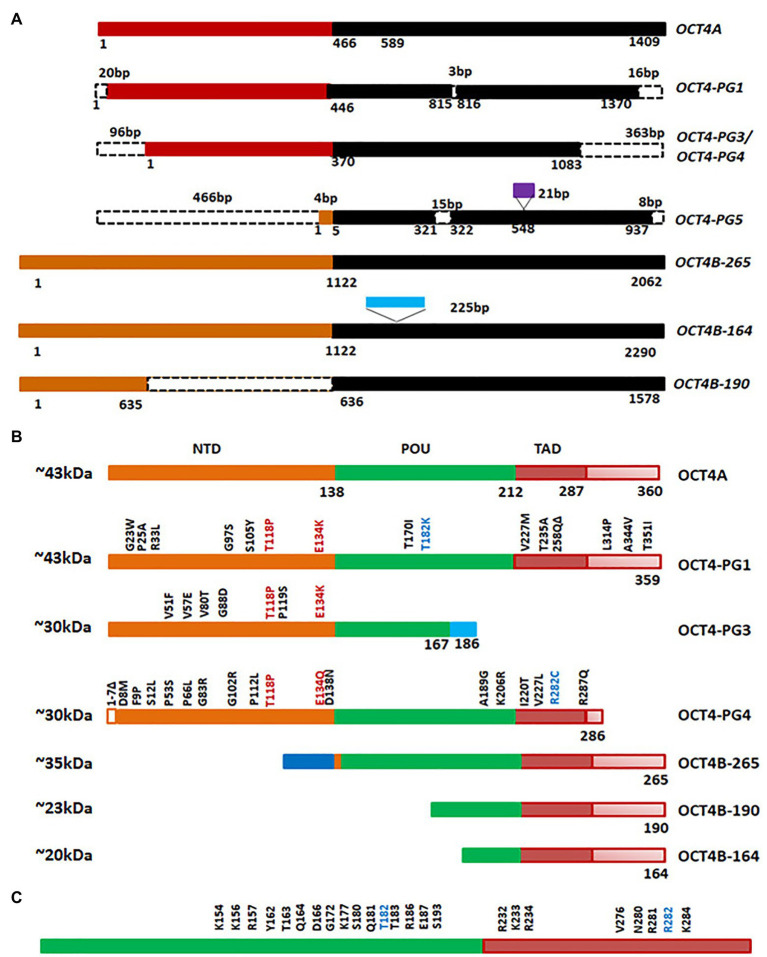
Octamer binding transcriptional factor 4 (OCT4) variants in cancer. **(A)** The different isoforms and pseudogenes of OCT4 expressed in cancer at the RNA level. The conserved regions are shown in the same color. The dotted lines indicate absence of the region. The sequences inserted are shown in purple or blue boxes. **(B)** Protein expression of the variants. Conserved amino acid stretches are shown by the same color. The point mutations in the pseudogenes are shown in the figure. **(C)** The important residues of human OCT4A (POU and TAD domain) that are critical for self-renewal.

**Figure 6:**
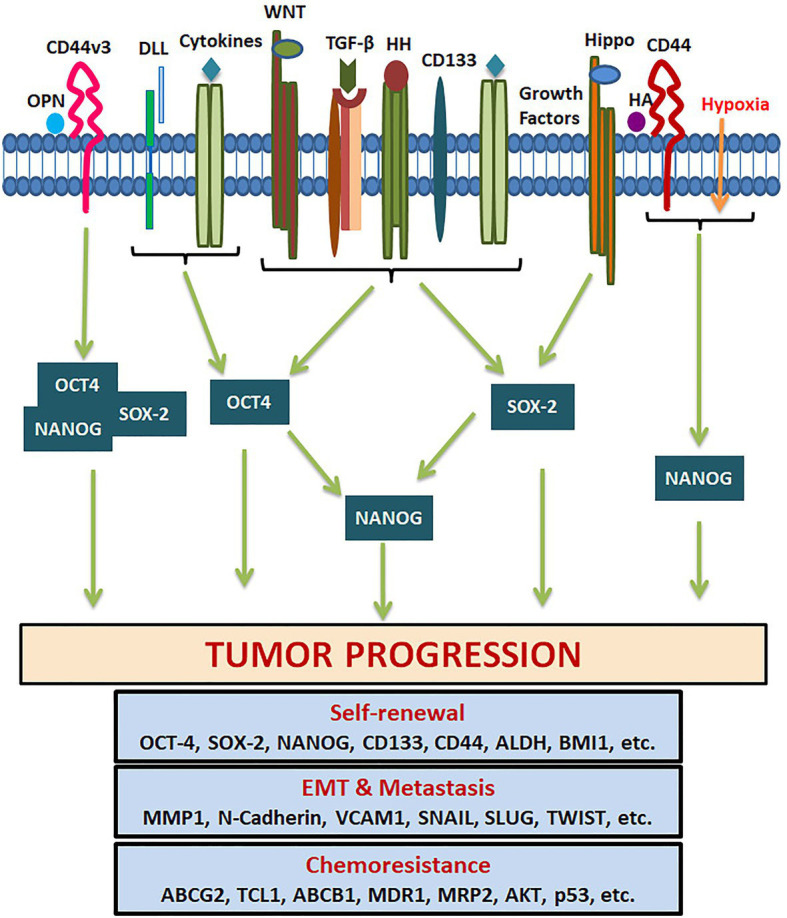
Pluripotency genes in the regulation of tumor properties. Several tumor microenvironment factors and extracellular matrix components activate different signaling pathways that regulate the expression of OCT4, SOX-2, and NANOG. They either act alone or together for the transcriptional activation of genes responsible for CSC self-renewal, EMT, metastasis, cell survival, and chemoresistance.

Octamer binding transcriptional factor 4 (OCT4, OCT3, and OCT3/4), encoded by *POU5F1* gene, is the master regulator of pluripotency in embryonic stem cells. The functional *POU5F1* gene is located on chromosome 6 in humans, while six different pseudogenes of it are located at different chromosomes-*POU5F1P1*, *P2*, *P3*, *P4*, *P5*, and *P6* (Villodre et al., 2016). *POU5F1* gene is transcribed into three mRNA forms, *OCT4A*, *OCT4B*, and *OCT4B1*. The different isoforms of OCT4 and the pseudogene products at the mRNA level and protein level are represented in Figures 5A,B. The critical residues of OCT4A important for self-renewal property are marked in Figure 5C. As shown in the figure, detection of OCT4 by RT-PCR or antibody will give results of different pseudogenes and isoforms together. Different isoforms of OCT4 (OCT4A, B and B1) are coming under the regulation of the same promoter, and are regulated by alternative splicing of exons. While OCT4A regulates self-renewal property, OCT4B and OCT4B1 control stress response (Guo et al., 2012). These isoforms expressed in the stem cell population respond to stress conditions, and their expression is not limited to stem cells (Guo et al., 2012). High OCT4 expression is a marker for poor prognosis; aggressiveness, short over-all survival and chemo-resistance, in several malignancies, and thus it is considered as a CSC marker (Table 1; Mohiuddin et al., 2020). Although the expression of OCT4A is shown in some cancers, its relevance is doubtful, since the products of pseudogenes were not considered in the experimental design (De Resende et al., 2013; Asadi et al., 2016; Soheili et al., 2017). OCT4B and B1 forms are over-expressed in various malignancies and consistent with their anti-proliferative effect and anti-apoptotic function, its expression is correlated to aggressiveness of the tumor (Asadi et al., 2011, 2016; Gazouli et al., 2012; De Resende et al., 2013; Soheili et al., 2017). Pseudogenes, like OCT4-PG1, are also implicated in cancer as the over-expression of this gene promotes tumorigenicity *in vitro* and *in vivo*, consistent with the association of OCT4-PG1 over-expression and poor prognosis in gastric cancer (Hayashi et al., 2015). In spite of the confusion regarding the isoforms and pseudogenes of OCT4 expressed in CSCs, many studies show the importance of the molecule in CSC characteristics (Zhang et al., 2020). The signaling event leading to the regulation of OCT4 expression and its downstream targets, regulating CSC characteristics, are summarized in Figure 6.

SOX2 is a transcriptional factor involved in embryonic development and generation of iPSCs, which controls the expression of genes required for maintenance of pluripotency and self-renewal (Chan et al., 2011). NANOG is a homeo-box binding transcriptional factor essential for the maintenance of pluripotency and self-renewal of embryonic stem cells, being one of the downstream targets of OCT4 and SOX2. The downstream signaling of pluripotency genes regulating CSC characteristics are summarized in Figure 6. *NANOG* has two transcript variants coding for two isoforms performing the same function with comparable efficiency. Even though *NANOG* is silenced in normal somatic cells, an aberrant expression is reported in a wide variety of cancers, correlating to poor survival (Yang et al., 2020). During the early stages of embryogenesis, NANOG prevents the activation of the BMP pathway to block differentiation. Even though *NANOG* undergoes auto-repression with differentiation, its aberrant overexpression leads to increased proliferation by entering into S-phase (Yu and Cirillo, 2020).

Hypoxia, one of the factors that induce the expression of pluripotency markers, is shown to enrich a Quiescent population, characterized by the high expressions of *POU5F1*, *SOX2*, and *NANOG*; low expression of proliferative markers (*CCNA2* and *MKI67*) and EMT marker like *ID1*, when analyzed by single-cell RNA-Seq using breast cancer cells (Akrap et al., 2016). When there is a transition of this Quiescent population to a Progenitor-like population, the proliferative markers and ID1 increase with a concomitant down-regulation of *POU5F1*, *SOX2*, and *NANOG* (Akrap et al., 2016).

## Other Molecules as CSC Markers

### ALDH1A1

Aldehyde dehydrogenase comprises a family of enzymes including 19 subtypes that converts aldehydes to their corresponding carboxylic acids to prevent oxidative stress in cells. They are located at different chromosomal loci controlled by unique promoters, and they may localize to different cellular compartments like cytoplasm, mitochondria, nucleus, and endoplasmic reticulum. Among the different isoforms, the cytoplasmic variants are responsible for the retinoic acid (RA) biosynthesis, which is a critical molecule involved in retinoic acid receptor (RAR) signaling, regulating stemness. Though retinol can be oxidized by any of the cytosolic ALDH to retinaldehyde, its irreversible conversion to RA requires specific ALDH isozymes ALDH1A1, ALDH1A2, ALDH1A3, or ALDH8A1 (Zhao et al., 1996; Elizondo et al., 2000; Marchitti et al., 2008). These isoforms that can convert retinaldehyde to RA are associated with stem cells of normal and cancer origin. ALDH1A1 was first identified as a stemness marker in hematopoietic stem cells and neural stem cells (Storms et al., 1999; Corti et al., 2006). Later, when the hypothesis of CSCs emerged, it was recognized as a CSC marker in a wide variety of cancers (Vassalli, 2019). Different oncogenic signaling including TGF-β, Notch, and WNT pathways and feedback activation by RA signaling is shown to regulate the expression of ALDH1A1 (Tomita et al., 2016). Apart from the RAR signaling, ALDH1A1 promotes a self-renewing population through tumor growth, self-protection by anti-oxidant activity and the development of drug resistance by its catalytic potential (Tomita et al., 2016). All these factors point out that ALDH1A1 is also a universal marker for the self-renewing population.

ALDH^Hi^ cells are considered as stem cells capable of forming mammospheres in normal and malignant breast tissue. The heterogeneity within normal mammary ALDH^Hi^ cells were evaluated by single-cell RNA-Seq from normal human breast samples (Colacino et al., 2018). The study revealed that ALDH^Hi^ cells that co-express CD44 have a different gene signature from cells that do not express CD44. The dual positive cells have high expression of *SOX2*, and EMT markers *ID1*, *TWIST*, and *VIM* (Colacino et al., 2018). ALDH^Hi^ cells segregate to four clusters, where cluster 1 had gene expression in low amount in general with a modest amount of stemness markers *ALDH1A3*, *CD44*, and *SOX2*, epithelial marker *PGR*, and EMT markers *ID1* and *VIM*, probably denoting a Quiescent population. Cluster 2 showed CSC features with high expression of stemness markers *ALDH1A3*, *CD44*, *CD133*, *ITGA6*, and *SOX2*, epithelial marker *CDH1*and *EPCAM*, and EMT markers *ID1* and *VIM*, denoting a hybrid EMT phenotype. Cluster 3 and Cluster 4 were representing epithelial and mesenchymal phenotype, respectively. In Cluster 3, the subtype of ALDH was ALDH1A1 instead of ALDH1A3. In breast tissue, the expression of subtypes of ALDH is dependent on localization. ALDH1A1 cells localize in small lobules, while ALDH1A3 cells localize to extralobular ducts (Colacino et al., 2018). The significance of ALDH^Hi^ population expressing ALDH1A1 at the single-cell level was investigated in the cancer context also. In lung cancer, there is a subpopulation of ALDH1A1 cells, the population size of which increases progressively with the stage of the disease (Taverna et al., 2020). But this increase was not associated with acquisition of drug resistance (Taverna et al., 2020).

## Reporters for CSCs

Conventionally, CSCs are identified and isolated by fluorescent activated cell sorting (FACS) or magnetic activated cell sorting (MACS) using antibodies for cell surface markers. But most of the cell surface markers alone are not reliable markers for CSCs. The intracellular molecules like pluripotency markers, which are more reliable CSCs markers, cannot be used for isolating live CSCs using their antibodies, because the permeabilization for detection will kill the cells. An alternative way of detection of CSCs in cultured cells is by making reporters for the CSCs markers. In the majority of the cases, the reporters are made to read the promoter activity of the particular gene. In some cases, where the CSC marker itself is a transcription factor, the reporter can be made using the promoter of the target gene. So, it is evident that a reporter construct for a CSC marker is not possible if the function of the molecule is regulated post-translationally or the function is limited to certain splice variants. Thus reporters for CD133 and CD44 are not useful for marking CSCs. So far, there are reports for preclinical studies using reporters for OCT4, SOX2, NANOG, ALDH1A1, and ABCG2, which will be discussed in detail.

Since the expression of OCT4 is associated with stemness, chemoresistance, and metastatic property of cancer cells, several attempts were made to construct reporters for OCT4 to track the CSCs. One of the widely used reporters is phOCT4-EGFP, where the expression of EGFP is under the control of the human OCT4 promoter. In breast cancer, the cells that express high EGFP (OCT4^hi^) mark highly immature cell population possessing self-renewal ability, quiescence, asymmetric division, long doubling time, and high metastatic and invasive capacity (Patel et al., 2012). It is also shown to mark dormant breast cancer cells residing in bone marrow, which are responsible for metastasis and tumor recurrence (Bliss et al., 2016). The relevance of this reporter in tracking MICs was shown in osteosarcoma and colorectal cancer also (Levings et al., 2009; Fujino and Miyoshi, 2019). It might also be useful to identify drug-resistant CSCs, as OCT4^hi^ cells were shown to be enriched in the sorafenib-resistant population in liver cancer (Wu et al., 2015). Taken together, the activation of *POU5F1* promoter, irrespective of the isoforms expressed, is an indication of dormancy in CSCs, which might lead to drug resistance or metastasis.

Although SOX2 can be considered as a robust marker for CSCs, a promoter-based reporter for SOX2 is not advisable in several malignancies. The gene amplification of *SOX2* is leading to the over-expression of the molecule. It is not resulted by the hyperactivity of a single promoter, but a cumulative activation of multiple promoters (Zhang et al., 2012). So, for analyzing SOX2 hyperactivation, another kind of reporter is widely used. SOX2 SRR2 pGreenFire Response Reporter is made by taking SOX2 regulatory region 2 (SRR2), a consensus DNA sequence seen on SOX2 target genes, to drive GFP. Using this reporter, CSCs were identified in different cancers (Wu et al., 2012, 2018; Iglesias et al., 2014), and it is shown to mark CSCs with cisplatin resistance in breast cancer (Soleymani Abyaneh et al., 2018). SORE6-GFP is another reporter made to evaluate the transcriptional activity of SOX2 in complex with OCT4. Six tandem repeats of composite OCT4/SOX2 response element, derived from *NANOG* promoter was cloned upstream to EGFP fluorescent protein to generate this reporter (Tang et al., 2015). This reporter identifies CSCs with metastatic potential and chemoresistance in breast cancer (Tang et al., 2015), gastric cancer (Padua et al., 2020), and prostate cancer (Vaddi et al., 2019).

Since NANOG is considered as a *bona fide* CSC marker, *NANOG* promoter-driven GFP is used to mark CSCs of different origins. NANOG-GFP was useful in characterizing CSCs of triple-negative breast cancer (Thiagarajan et al., 2015) and ovarian cancer (Wiechert et al., 2016). Similar NANOG reporters were constructed later by other groups (Buczek et al., 2018; Wei et al., 2018). A lentiviral NANOG-GFP reporter expressing luciferase was shown to mark drug-resistant CSCs in colorectal cancer, both *in vitro* and in mouse xenograft models (Wei et al., 2018).

As ALDH1A1 is a CSC marker for a wide variety of cancers, reporter constructs for CSCs based on ALDH1A1 promoter have been generated (Gener et al., 2015; Shanmugam et al., 2019). An ALDH1A1 promoter-driven tdTomato is reported for evaluating the drug sensitivity in breast cancer and colon cancer cell lines (Gener et al., 2015). Recently, we reported a similar construct, ALDH1A1-DsRed2, for marking CSCs of oral cancer (Shanmugam et al., 2019).

The expression of ABCG2, at the transcriptional level, is regulated by an epigenetic mechanism. It has been shown that the acquisition of multidrug-resistant phenotype by the upregulation of ABCG2 is achieved by the reversal of promoter methylation (Bram et al., 2009). Since transcriptional regulation, at least in part, is driven by epigenetic mechanisms, the stable expression of a reporter driven by the ABCG2 promoter will not be useful for identification of the drug-resistant CSCs. So, a reporter cell line is recently made using CRISPR-Cas9 gene editing coupled with the homology-directed repair. They targeted the EGFP coding sequence to the translational start site of ABCG2, generating ABCG2 knock-out and *in situ* tagged ABCG2 reporter cells (Kovacsics et al., 2020). This fluorescent reporter system allowed the detection of endogenous regulation of ABCG2 expression by different stress responses and offers a method to screen molecules that can inhibit drug-resistant CSCs.

## The Clinical Relevance of Markers and Reporters

Considering the importance of CSCs in prognosis, there were many attempts to target CSCs at the preclinical and clinical level (Saygin et al., 2019). The different approaches to target CSCs were using drugs that (a) inhibit self-renewal pathways like Notch, hedgehog (HH), and WNT (b) target CSC niche, and (c) block drug transporters (Saygin et al., 2019). Another important strategy is immunotherapy (Saygin et al., 2019). Though some drugs were successful in both preclinical and clinical trials, there were many other drugs that proved successful in preclinical studies *in vitro*, but did not prove efficient in clinical studies (Saygin et al., 2019). Vismodegib is an antagonist for HH signaling that efficiently decreased CSCs of pancreatic cancer and lung cancer *in vitro* (Singh et al., 2011; Ahmad et al., 2013). When this molecule was tested in colorectal cancer patients, there was no significant benefit observed for the drug (Berlin et al., 2013).

One of the reasons for the unsuccessful translation of CSC targeting drugs is the inefficient detection of CSCs in preclinical screening. At present, CSCs are characterized by functional properties, like sphere formation efficiency and tumor initiation potential in serial dilutions and by using different phenotypic markers. Conventionally, we evaluate a drug for its efficacy in reducing CSCs based on the expression profile of markers, which do not identify all the CSCs. As we have discussed earlier (Figure 2), a combination of pluripotency marker and ALDH1A1/3 or ABCG2 will identify all the CSC sub-populations. Since an ideal CSC targeting drug should deplete all the heterogeneous CSCs, a screening strategy should include these marker profiles. Additionally, all these CSC subpopulations are maintained by different signaling pathways and hence they show differences in their responses to chemotherapeutic drugs. If we identify the critical molecules or pathways that are ideal for targeting each population, we might achieve good therapeutic outcome. Further, if we can use reporter constructs, it will be extremely useful not only for the isolation of these population, but also for designing drug screening strategies and real-time monitoring of these population *in vitro* and *in vivo*.

## Conclusion and Persepectives

All the CSC populations can initiate a tumor but the drug resistance and metastasis initiating capacity might be exhibited by cells in the Progenitor-like stage. The proliferating cells exhibiting high hybrid EMT might also be important in metastasis, even though they lack the tumor-initiation potential. These cells can migrate to new sites and reside as dormant cells, which might have been the dormant population observed in label-retaining cells with low expression of all the genes, including the pluripotency markers. These cells may later acquire tumor-initiation potential, since all these populations exhibit plasticity (Figure 2). Among the different populations described, this high hybrid EMT dormant population is the least characterized, and warrants more studies to understand their biology to come up with a reliable marker for this population.

If we use a single pluripotency marker to screen drugs targeting CSCs, we identify the Quiescent and Progenitor population, but do not take the Progenitor-like population into account, which are really important with respect to drug response and metastasis. Even though they are proliferating cells, they show chemoresistance attributed to the expression of drug resistance proteins like ABCG2 and ALDH. On the other hand, if we use ALDH1A1 or ABCG2, we miss the Quiescent and Progenitor population, which remain dormant and cause recurrence. So, an ideal marker profile to use for drug screening will be a combination of pluripotency markers, CD44, CD133, ALDH1A1, and ABCG2, where we can pick up all the heterogeneous CSCs including Quiescent CSC, Progenitor CSC, Progenitor-like CSC, and MIC (Figure 2). Among these markers, CD44 and CD133 reporters are not relevant as their function is not regulated at the transcriptional level. Though ABCG2 reporters are available, they are not useful in CSCs because the expression is regulated by the promoter demethylation in cancer. Hence, we propose that a dual reporter including one of the pluripotency markers (phOCT4-EGFP/NANOG-GFP/SORE6-GFP/SOX2 SRR2-pGreen fire) and ALDH1A1-DsRed2 can efficiently mark all the heterogeneous CSCs, at least in breast cancer. Though this system does not distinguish between drug-resistant CSCs and MICs, a low pluripotency marker/high ALDH1A1 population will include both the subsets. Another shortcoming of the system is that it will not mark the proliferative non-CSCs possessing hybrid EMT. This system can be easily adapted for high-throughput screening of CSC-targeting drugs. A successful CSC-targeting drug should eradicate all the different pools of CSCs. The proliferative population with high hybrid EMT probably will be eliminated by standard chemotherapeutic drugs targeting proliferating cells. Still, a population that gets converted to dormant stage poses a threat to the treatment outcome. Since we know that functional diversity exists in these heterogeneous populations, the response of these populations to drugs will be different. More studies are required to identify specific molecular targets that can be used for drug development to abolish all the relevant populations that lead to recurrence. All these observations and conclusions of the marker profiles to identify different subpopulations are based on studies on breast cancer. Whether these marker profiles will be applicable to other forms of cancer is yet to be explored.

## Author Contributions

AM performed the analysis, interpreted the data, and drafted the manuscript. RR, PK, and GM contributed critically for important intellectual content of the manuscript. TM conceptualized and designed the study and revised the manuscript critically. All authors contributed to the article and approved the submitted version.

### Conflict of Interest

The authors declare that the research was conducted in the absence of any commercial or financial relationships that could be construed as a potential conflict of interest.
